# Supplementary material for: Cryo-EM analyses reveal the common mechanism and diversification in the activation of RET by different ligands
Source: eLife. 2019 Sep 19;8:e47650. doi: 10.7554/eLife.47650 (PMC6760901; doi:10.7554/eLife.47650)
Supplement: Supplementary file 1. [file elife-47650-supp1.docx]

>Ret-29-635-His

LYFSRDAYWEKLYVDQAAGTPL

LYVHALRDAPEEVPSFRLGQHLYGTYRTRLHENNWICIQEDTGLLYLNRS

LDHSSWEKLSVRNRGFPLLTVYLKVFLSPTSLREGECQWPGCARVYFSFF

NTSFPACSSLKPRELCFPETRPSFRIRENRPPGTFHQFRLLPVQFLCPNI

SVAYRLLEGEGLPFRCAPDSLEVSTRWALDREQREKYELVAVCTVHAGAR

EEVVMVPFPVTVYDEDDSAPTFPAGVDTASAVVEFKRKEDTVVATLRVFD

ADVVPASGELVRRYTSTLLPGDTWAQQTFRVEHWPNETSVQANGSFVRAT

VHDYRLVLNRNLSISENRTMQLAVLVNDSDFQGPGAGVLLLHFNVSVLPV

SLHLPSTYSLSVSRRARRFAQIGKVCVENCQAFSGINVQYKLHSSGANCS

TLGVVTSAEDTSGILFVNDTKALRRPKCAELHYMVVATDQQTSRQAQAQL

LVTVEGSYVAEEAGCPLSCAVSKRRLECEECGGLGSPTGRCEWRQGDGKG

ITRNFSTCSPSTKTCPDGHCDVVETQDINICPQDCLRGSIVGGHEPGEPR

GIKAGYGTCNCFPEEEKCFCEPEDIQDPLCDELCRGTHHHHHHHH-

>GDF15-197-end

MGSSHHHHHHSQDLEVLFQGPGSARNG

DHCPLGPGRC CRLHTVRASL EDLGWADWVL SPREVQVTMC IGACPSQFRA

ANMHAQIKTS LHRLKPDTVP APCCVPASYN PMVLIQKTDT GVSLQTYDDL

LAKDCHCI

>GFRAL-20-352

QTNNCTYLREQCLRDANGCKHAWRVMEDACNDSDPGDPCKM

RNSSYCNLSIQYLVESNFQFKECLCTDDFYCTVNKLLGKKCINKSDNVKEDKFKWNLTTR

SHHGFKGMWSCLEVAEACVGDVVCNAQLASYLKACSANGNPCDLKQCQAAIRFFYQNIPF

NIAQMLAFCDCAQSDIPCQQSKEALHSKTCAVNMVPPPTCLSVIRSCQNDELCRRHYRTF

QSKCWQRVTRKCHEDENCISTLSKQDLTCSGSDDCKAAYIDILGTVLQVQCTCRTITQSE

ESLCKIFQHMLHRKSCFNYPTLSNVKGMALYTRKHANKITLTGFHSPFNGEVGTHHHHHHHH-

>GFRA1-25-426-His

DRLDCVKASDQCLKEQSCSTKYRTLR

QCVAGKETNFSLASGLEAKDECRSAMEALKQKSLYNCRCKRGMKKEKNCL

RIYWSMYQSLQGNDLLEDSPYEPVNSRLSDIFRVVPFISDVFQQVEHIPK

GNNCLDAAKACNLDDICKKYRSAYITPCTTSVSNDVCNRRKCHKALRQFF

DKVPAKHSYGMLFCSCRDIACTERRRQTIVPVCSYEEREKPNCLNLQDSC

KTNYICRSRLADFFTNCQPESRSVSSCLKENYADCLLAYSGLIGTVMTPN

YIDSSSLSVAPWCDCSNSGNDLEECLKFLNFFKDNTCLKNAIQAFGNGSD

VTVWQPAFPVQTTTATTTTALRVKNKPLGPAGSENEIPTHVLPPCANLQA

QKLKSNVSGNTHLCISNGNYEKEGLGGTHHHHHHHH-

>GDNF-77-211

MGSSHHHHHHSQDLEVLFQGPGS

RSPDKQMAVLPRRERNRQAAAANPENS

RGKGRRGQRGKNRGCVLTAIHLNVTDLGLGYETKEELIFRYCSGSCDAAE

TTYDKILKNLSRNRRLVSDKVGQACCRPIAFDDDLSFLDDNLXYHILRKH

SAKRCGCI-

>GFRA2-

SSLQGPELHGWRPPVDCVRANELCAAESNCSSRYRTLRQCLAGRDRN

TMLANKECQAALEVLQESPLYDCRCKRGMKKELQCLQIYWSIHLGLTEGEEFYEASPYEPVTSRLSDIFR

LASIFSGTGADPVVSAKSNHCLDAAKACNLNDNCKKLRSSYISICNREISPTERCNRRKCHKALRQFFDR

VPSEYTYRMLFCSCQDQACAERRRQTILPSCSYEDKEKPNCLDLRGVCRTDHLCRSRLADFHANCRASYQ

TVTSCPADNYQACLGSYAGMIGFDMTPNYVDSSPTGIVVSPWCSCRGSGNMEEECEKFLRDFTENPCLRN

AIQAFGNGTDVNGTHHHHHHHH

>NRTN-95-197

MGSSHHHHHHSSGLVPRGSHMASMSDSEVNQEAKPEVKPEVKPETHINLK

VSDGSSEIFFKIKKTTPLRRLMEAFAKRQGKEMDSLRFLYDGIRIQADQT

PEDLDMEDNDIIEAHREQIGGS

RARLGARPCGLRELEVRVSELGLGYASDETVLFRYCAGACEAAARV

YDLGLRRLRQRRRLRRERVRAQPCCRPTAYEDEVSFLDAHSRYHTVHELSARECACV

>GFRA3-32-363

DPLPTESRL MNSCLQARRK

CQADPTCSAA YHHLDSCTSS ISTPLPSEEP SVPADCLEAA QQLRNSSLIG

CMCHRRMKNQ VACLDIYWTV HRARSLGNYE LDVSPYEDTV TSKPWKMNLS

KLNMLKPDSD LCLKFAMLCT LNDKCDRLRK AYGEACSGPH CQRHVCLRQL

LTFFEKAAEP HAQGLLLCPC APNDRGCGER RRNTIAPNCA LPPVAPNCLE

LRRLCFSDPL CRSRLVDFQT HCHPMDILGT CATEQSRCLR AYLGLIGTAM

TPNFVSNVNT SVALSCTCRG SGNLQEECEM LEGFFSHNPC LTEAIAAKMR

FHSQLFSQDW PHPGTHHHHHHHH-

>ARTN-108-220

MGSSHHHHHHSSGLVPRGSHMASMSDSEVNQEAKPEVKPEVKPETHINLK

VSDGSSEIFFKIKKTTPLRRLMEAFAKRQGKEMDSLRFLYDGIRIQADQT

PEDLDMEDNDIIEAHREQIGGS AGGPGSRARAAGARGCRLRSQLVPVRALGLGHRSDELVRFRFCSGSCRRARSPHDLSLASLLGAGALRPPPGSRPVSQPCCRPTRYEAVSFMDVNSTWRTVDRLSATACGCLG
